# High frequency of mutations in 'dyshormonogenesis genes' in severe congenital hypothyroidism

**DOI:** 10.1371/journal.pone.0204323

**Published:** 2018-09-21

**Authors:** Nina Makretskaya, Olga Bezlepkina, Anna Kolodkina, Alexey Kiyaev, Evgeny V. Vasilyev, Vasily Petrov, Svetlana Kalinenkova, Oleg Malievsky, Ivan I. Dedov, Anatoly Tiulpakov

**Affiliations:** 1 Department and Laboratory of Inherited Endocrine Disorders, Endocrinology Research Centre, Moscow, Russian Federation; 2 Department of Polyclinic Pediatrics, Ural State Medical University, Ekaterinburg, Russian Federation; 3 Genetics Laboratory, Moscow Regional Research and Clinical Institute, Moscow, Russian Federation; 4 Department of Hospital Pediatrics, Republican Children’s Clinical Hospital, Ufa, Russian Federation; University Claude Bernard Lyon 1, FRANCE

## Abstract

**Objective:**

Results of the screening of disease causative mutations in congenital hypothyroidism (CH) vary significantly, depending on the sequence strategy, patients’ inclusion criteria and bioinformatics. The objective was to study the molecular basis of severe congenital hypothyroidism, using the next generation sequencing (NGS) and the recent guidelines for assessment of sequence variants.

**Design:**

243 patients with CH (TSH levels at neonatal screening or retesting greater than 90 mU/l) and 56 control subjects were included in the study.

**Methods:**

A custom NGS panel targeting 12 CH causative genes was used for sequencing. The sequence variants were rated according to American College of Medical Genetics and Genomics (ACMG) guidelines.

**Results:**

In total, 48 pathogenic, 7 likely pathogenic and 57 variants of uncertain significance were identified in 92/243 patients (37.9%), while 4 variants of uncertain significance were found in 4/56 control subjects (7.1%). 13.1% (12/92) of the cases showed variants in ‘thyroid dysgenesis’ (TD) genes: *TSHR*, n = 6; *NKX2-1*, n = 2; *NKX2-5*, n = 1; *PAX8*, n = 3. The variants in ‘dyshormonogenesis’ (DH) genes were found in 84.8% (78/92) of cases: *TPO*, n = 30; *DUOX2*, n = 24; *TG*, n = 8; *SLC5A5*, n = 3; *SLC26A4*, n = 6; *IYD*, n = 1. 8 patients showed oligonenic variants. The majority of variants identified in DH genes were monoallelic.

**Conclusions:**

In contrast to earlier studies demonstrating the predominance of TD in severe CH, the majority of variants identified in our study were in DH genes. A large proportion of monoallelic variants detected among DH genes suggests that non-mendelian mechanisms may play a role in the development of CH.

## Introduction

Congenital hypothyroidism (CH) is a partial or complete loss of function of the thyroid gland that affects infants from birth, being the most common inborn endocrine disorders, with a prevalence of 1 in 3000–4000 newborns [1]. Historically, insights into the etiology of CH were given by the results of scintigraphy and ultrasonography studies, according to which, thyroid dysgenesis (TD) was defined in 80–85% of patients, while the remaining 15–20% of cases were believed to be due to thyroid dyshormonogenesis (DH) [2,3]. At least 12 genes have been described that are involved in the pathogenesis of CH, part of which were shown to be involved in thyroid dysgenesis (TD) [4] (*TSHR* [5], *PAX8* [6], *NKX2-5* [7], *FOXE1* [8], *NKX2-1* [9,10]), while the others were linked to the defects in biosynthesis of thyroid hormones, i.e. dyshormonogenesis (DH) (*TPO* [11], *IYD* [12], *SLC26A4* [13], *TG* [14], *SLC5A5* [15], *DUOX2* [16], *DOUXA2* [17]) [18]. Studies on the molecular basis of CH in the pre-NGS era were usually performed in patients with specific clinical or thyroid imaging characteristics and were focused on a limited number of genes and (or) a small number of cases. Such studies revealed molecular origin of CH in less than 10% of cases [19–22]. The introduction of the next generation sequencing (NGS) made the studies in CH more efficient and showed a higher rate of mutations in subjects with CH [23–28].

In the current paper we present results of NGS in 243 Russian patients with CH. In this study covering the largest patients’ cohort reported to date we have included cases only with severe CH (TSH at diagnosis > 90 mU/L). Assessment of pathogenicity of sequence variants was based on the American College of Medical Genetics and Genomics (ACMG) guidelines [29], which eliminated from analysis single nucleotide variants with minor allele frequency (MAF) greater than 0.001.

## Subjects and methods

### Subjects

This study was approved by the local ethics committee of the Endocrinology Research Centre (Protocol №12 dated 22.10.2014). Informed written consents were obtained from the patients or (and) the parents.

243 patients (94 males, 149 females) with severe CH, defined as TSH levels at neonatal screening or re-testing greater than 90 mU/L, were included in the study. At the time of the study the age of the patients ranged from 4 weeks to 18 years (median, 4.5 years).

56 subjects (24 males, 32 females) were included in the control group. The inclusion criteria were normal levels of TSH and free T4, no thyroid antibodies, no changes according to the thyroid ultrasound.

### DNA sequencing

Genomic DNA was extracted from peripheral leukocytes using PureLink® Genomic DNA Mini Kits (Thermo Scientific, USA). A custom Ion Ampliseq™ panel (Ion Torrent, Thermo Scientific, USA) targeting 12 genes associated with hypothyroidism (*TPO*, *PAX8*, *NKX2-5*, *IYD*, *SLC26A4*, *TG*, *FOXE1*, *NKX2-1*, *DUOX2*, *DOUXA2*, *TSHR*, *SLC5A5*) was used for DNA library preparation. Sequencing was performed using Personal Genome Machine (PGM) semiconductor sequencer (Ion Torrent, Thermo Scientific, USA). Bioinformatics analysis was carried out using Torrent Suite 4.2.1 (Thermo Scientific, USA) and ANNOVAR ver. 2014Nov12 software packages [30]. The results of the NGS were confirmed by Sanger sequencing using Genetic Analyzer 3130 sequencer (Life Technologies, USA). Interpretation of the sequencing results and assessment of the pathogenicity of sequence variants were performed according to the ACMG guidelines [29]. Sequence variants rated as ‘benign’ or ‘likely benign’ were excluded from the analysis. A description of the sequence variants was carried out in accordance with the recommendations of den Dunnen and Antonarakis [31].

### MLPA

A multiplex ligation-dependent probe amplification (MLPA) was carried out on 24 patients: one patient with suspected deletion of multiple exons in *PAX8* gene, as determined by the NGS coverage analysis; and 23 patients with a single heterozygous mutation in *TPO* or *TSHR* genes. SALSA MLPA probemix P319 set (genes *TPO*, *PAX8*, *FOXE1*, *NKX2-1* and *TSHR*, MRC-Holland, Netherlands) and a standard set of reagents SALSA MLPA EK1–FAM (MRC-Holland, Netherlands) were used. Data processing was carried out using software Coffalyser.Net (MRC-Holland, Netherlands).

### Statistical analysis

Pearson χ2 and odds ratio were applied to analyze the results of the study.

## Results

**Fig 1:**
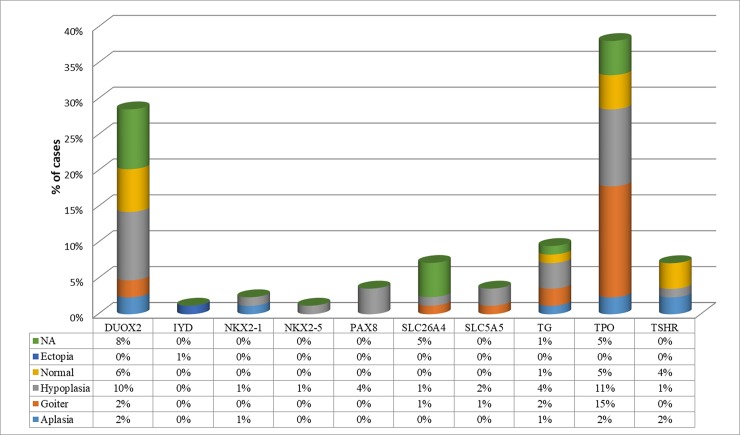
Percent distribution of monogenic variants identified in genes according to the CH phenotype.

NGS identified 63 different sequence variants in 92 of 243 patients (37.9%). Homozygous variants were identified in 12.0% (11/92), compound heterozygous variants in 13.0% (12/92), heterozygous variants in 66.3% (61/92), 8.7% variants were identified in two genes (8/92). 84.8% (78/92) variants were in the DH genes (*TPO*, *IYD*, *SLC26A4*, *TG*, *SLC5A5*, *DUOX2*, *DOUXA2*), 13.1% (12/92) of the variants were identified in the TD genes (Fig 1). Variants in two groups of genes were identified in 2 patients (2.1%, 2/92).

In our study the majority of variants were found in *TPO* gene (in 30 of 92 patients, 32.6%) (Table 1). Defects in *TPO* gene included insertions and deletions with frameshift (n = 4), nonsense variants (n = 1), missense variants (n = 13), splice-site variants (n = 2). No deletions or insertions were identified in patients from this group using MLPA. The second most frequent findings were changes in *DUOX2* gene (26.1%, 24/92), we identified a deletion with frameshift, 2 nonsense and 7 missense variants (Table 2). 8 patients (8.7%) showed variants in *TG* gene. The range of variants in *TG* included missense (n = 5), nonsense (n = 1), and splicing (n = 1) (Table 1). In total, variants in *SLC5A5*, *SLC26A4* and *IYD* genes were detected in 10 patients (10.9%) (Table 1).

**Table 1:** Summary of nucleotide variants in DH genes, characteristics and clinical manifestations.

Subjects	Gene	NT alteration	AA alteration	Pathogenicity	Zygosity	ExAC*	gnomAD^	HGMD^#^	Thyroid gland	Assocaited abnormalities
**N1**	***TPO***	c.1181_1182insCGGC	p.A397PfsX76	**P**	Het	NA	0.000523	NA	hypoplasia	None
**N2**	***TPO***	c.1181_1182insCGGC	p.A397PfsX76	**P**	Het	NA	0.000523	NA	goiter	None
**N3**	***TPO***	c.1181_1182insCGGC	p.A397PfsX76	**P**	Het	NA	0.000523	NA	multinodular goiter	None
**N4**	***TPO***	c.1181_1182insCGGC	p.A397PfsX76	**P**	Het	NA	0.000523	NA	NA	None
**N5**	***TPO***	c.1181_1182insCGGC	p.A397PfsX76	**P**	Het	NA	0.000523	NA	goiter	None
**N6**	***TPO***	c.1851delC	p.S617RfsX23	**P**	Het	NA	NA	NA	goiter	None
**N7**	***TPO***	c.2618+1G>T		**P**	Het	NA	NA	NA	hypoplasia	None
**N8**	***TPO***	c.A1898T	p.D633V	US	Het	NA	NA	NA	goiter	None
**N9**	***TPO***	c.C1449A	p.N483K	US	Het	NA	NA	NA	aplasia	None
**N10**	***TPO***	c.C265T	p.R89X	**P**	Het	NA	0.000008	Reported [43]	hypoplasia	None
**N11**	***TPO***	c.C443T	p.A148V	US	Het	0.000049	0.000043	NA	aplasia	None
**N12**	***TPO***	c.G1581T	p.W527C	**LP**	Het	NA	0.000069	Reported [22]	goiter	None
**N13**	***TPO***	c.G1751A	p.R584Q	US	Het	0.000082	0.000072	NA	hypoplasia	None
**N14**	***TPO***	c.G1994A	p.R665Q	**LP**	Het	0.000025	0.000024	Reported [44]	goiter	None
**N15**	***TPO***	c.G2017A	p.E673K	US	Het	0.00011	0.00009	NA	hypoplasia	None
**N16**	***TPO***	c.G2017A	p.E673K	US	Het	0.00011	0.00009	NA	hypoplasia	None
**N17**	***TPO***	c.G2017A	p.E673K	US	Het	0.00011	0.00009	NA	NA	None
**N18**	***TPO***	c.G2017A	p.E673K	US	Het	0.00011	0.00009	NA	NA	None
**N19**	***TPO***	c.T289C	p.S97P	US	Het	NA	NA	NA	goiter	None
**N20**	***TPO***	c.C208G	p.P70A	US	Het	0.00071	0.00086	NA	Normal	None
**N21**	***TPO***	c.T289C	p.S97P	US	Het	NA	NA	NA	hypoplasia	None
**N22-1**	***TPO***	c.G1042A	p.G348R	US	ComHet	NA	NA	NA	hypoplasia	None
	***TPO***	c.G1465A	p.A489T	US		NA	0.000037	NA		
**N22-2**	***TPO***	c.G1042A	p.G348R	US	ComHet	NA	NA	NA	hypoplasia	None
	***TPO***	c.G1465A	p.A489T	US		NA	0.000037	NA		
**N23**	***TPO***	c.1851delC	p.S617RfsХ23	**P**	ComHet	NA	NA	NA	Normal	None
	***TPO***	c.2422delT	p.C808AfsX24	**P**		NA	0.000016	NA		
**N24**	***TPO***	c.2422delT	p.C808AfsX24	**P**	ComHet	NA	0.000016	NA	NA	None
	***TPO***	c.C208G	p.P70A	US		0.00071	0.00086	NA		
**N25**	***TPO***	c.C265T	p.R89X	**P**	ComHet	NA	0.000008	Reported [43]	multinodular goiter	Sensorineural hearing loss
	***TPO***	c.1181_1182insCGGC	p.A397PfsX76	**P**		NA	0.000523	NA		
**N26**	***TPO***	c.T391C	p.S131P	**LP**	ComHet	0.000058	0.000049	Reported [45]	multinodular goiter	None
	***TPO***	c.2386+2T>G		**LP**		NA	NA	NA		
**N27-1**	***TPO***	c.667_669delGAT	p.D223del	**P**	ComHet	NA	NA	NA	goiter	None
	***TPO***	c.2422delT	p.C808AfsX24	**P**		NA	0.000016	NA		
**N27-2**	***TPO***	c.667_669delGAT	p.D223del	**P**	ComHet	NA	NA	NA	goiter	None
	***TPO***	c.2422delT	p.C808AfsX24	**P**		NA	0.000016	NA		
**N28**	***TPO***	c.T281C	p.M94T	US	ComHet	NA	0.000007	NA	goiter	None
	***TPO***	c.A719T	p.D240V	US		NA	NA	NA		
**N29**	***DUOX2***	c.2895_2898del	p.S965fsX30	**P**	Het	0.0029	NA	Reported [46]	hypoplasia	None
**N30**	***DUOX2***	c.2895_2898del	p.S965fsX30	**P**	Hom	0.0029	NA	Reported [46]	goiter	None
**N31**	***DUOX2***	c.2895_2898del	p.S965fsX30	**P**	Het	0.0029	NA	Reported [46]	hypoplasia	None
**N32**	***DUOX2***	c.2895_2898del	p.S965fsX30	**P**	Het	0.0029	NA	Reported [46]	aplasia	None
**N33**	***DUOX2***	c.2895_2898del	p.S965fsX30	**P**	Hom	0.0029	NA	Reported [46]	NA	None
**N34**	***DUOX2***	c.2895_2898del	p.S965fsX30	**P**	Het	0.0029	NA	Reported [46]	hypoplasia	None
**N35**	***DUOX2***	c.2895_2898del	p.S965fsX30	**P**	Het	0.0029	NA	Reported [46]	Normal	None
**N36**	***DUOX2***	c.2895_2898del	p.S965fsX30	**P**	Hom	0.0029	NA	Reported [46]	hypoplasia	None
**N37**	***DUOX2***	c.2895_2898del	p.S965fsX30	**P**	Hom	0.0029	NA	Reported [46]	goiter	None
**N38**	***DUOX2***	c.2895_2898del	p.S965fsX30	**P**	Hom	0.0029	NA	Reported [46]	Normal	None
**N39**	***DUOX2***	c.2895_2898del	p.S965fsX30	**P**	Hom	0.0029	NA	Reported [46]	Normal	None
**N40**	***DUOX2***	c.2895_2898del	p.S965fsX30	**P**	Het	0.0029	NA	Reported [46]	hypoplasia	None
**N41**	***DUOX2***	c.2895_2898del	p.S965fsX30	**P**	Hom	0.0029	NA	Reported [46]	NA	None
**N42**	***DUOX2***	c.2895_2898del	p.S965fsX30	**P**	Het	0.0029	NA	Reported [46]	Normal	None
**N43**	***DUOX2***	c.2895_2898del	p.S965fsX30	**P**	Het	0.0029	NA	Reported [46]	Normal	None
**N44**	***DUOX2***	c.A4637G	p.E1546G	US	Het	0.00084	0.00081	NA	NA	None
**N45**	***DUOX2***	c.C1126T	p.R376W	US	Het	0.00012	0.00008	Reported [47]	aplasia	None
**N46**	***DUOX2***	c.C1294T	p.R432C	US	Het	NA	0.000004	NA	NA	None
**N47**	***DUOX2***	c.C3250T	p.R1084X	**P**	Het	0.000099	0.000087	NA	hypoplasia	None
**N48**	***DUOX2***	c.C3970T	p.P1324S	US	Het	NA	0.000008	NA	hypoplasia	None
**N49**	***DUOX2***	c.G1040A	p.R347K	US	Het	0.000034	0.000018	NA	NA	None
**N50**	***DUOX2***	c.A4637G	p.E1546G	US	Het	0.00084	0.00081	NA	hypoplasia	None
**N51**	***DUOX2***	c.T1366C	p.W456R	US	Het	NA	NA	NA	NA	
**N52**	***DUOX2***	c.2895_2898del	p.S965fsX30	**P**	ComHet	NA	NA	Reported [46]	NA	None
	***DUOX2***	c.C2056T	p.Q686X	**P**		NA	0.000004	Reported [46]		
**N53**	***TG***	c.5401+2T>C		**P**	Het	NA	NA	NA	goiter	None
**N54**	***TG***	c.C2338A	p.Q780K	US	Het	NA	NA	NA	hypoplasia	None
**N55**	***TG***	c.G1900A	p.G634R	US	Het	0.00049	0.0005	NA	aplasia	None
**N56**	***TG***	c.G2776T	p.E926X	**P**	Het	NA	NA	NA	goiter	None
**N57**	***TG***	c.G2977A	p.A993T	US	Het	0.00033	0.00039	NA	Normal	None
**N58**	***TG***	c.G2977A	p.A993T	US	Het	0.00033	0.00039	NA	hypoplasia	None
**N59**	***TG***	c.T2200A	p.S734T	US	Het	0.000017	0.000022	NA	hypoplasia	None
**N60**	***TG***	c.G455A	p.R152H	US	Het	0.00068	0.00072	NA	NA	None
**N61-1**	***SLC5A5***	c.C1906T	p.R636X	**P**	Hom	NA	0.000011	NA	hypoplasia	None
**N61-2**	***SLC5A5***	c.C1906T	p.R636X	**P**	Hom	NA	0.000011	NA	hypoplasia	None
**N62**	***SLC5A5***	c.469delA	p.N157fs	**P**	ComHet	NA	NA	NA	goiter	None
	***SLC5A5***	c.G1183A	p.G395R	**LP**		0.000066	0.000047	Reported [48]		
**N63**	***SLC26A4***	c.A1246C	p.T416P	**LP**	Het	0.00021	0.0002	Reported [49]	NA	None
**N64**	***SLC26A4***	c.A736C	p.N246H	US	Het	0.0000082	0.000004	NA	NA	Sensorineural hearing loss
**N65**	***SLC26A4***	c.G1483A	p.D495N	US	Het	NA	NA	NA	hypoplasia	None
**N66**	***SLC26A4***	c.G441A	p.M147I	US	Het	0.00051	0.0006060	Reported [50]	NA	None
**N67**	***SLC26A4***	c.G441A	p.M147I	US	Het	0.00051	0.0006060	Reported [50]	goiter	None
**N68**	***SLC26A4***	c.G2219T	p.G740V	US	Het	0.00027	0.00029	Reported [51]	NA	None
**N69**	***IYD***	c.C448T	p.R150X	**P**	Het	NA	0.000008	NA	ectopia	None

^**#**^ The Human Gene Mutation Database (HGMD® (http://www.hgmd.cf.ac.uk) [52]

*ExAC database (http://exac.broadinstitute.org) [36]

**^**gnomAD database (http://gnomad.broadinstitute.org/)

Pathogenicity: US, Uncertain significance; P, Pathogenic; LP, Likely pathogenic (pathogenicity rated according to ACMG guidelines [29], sequence variants rated as ‘benign’ or ‘likely benign’ were excluded from the analysis); NT, nucleotide; AA, amino acid; NA, not available; Het, heterozygous; ComHet, compound heterozygous; Hom, homozygous.

NCBI Reference Sequences (www.ncbi.nlm.nih.gov/nuccore): *TPO*, NM_000547; *DUOX2*, NM_014080; *TG*, NM_003235; *SLC5A5*, NM_000453; *SLC26A4*, NM_000441; *IYD*, NM_203395.

**Table 2:** Summary of nucleotide variants in TD genes, characteristics and clinical manifestations.

Subjects	Gene	NT alteration	AA alteration	Pathogenicity	Zygosity	ExAC*	gnomAD^	HGMD^#^	Thyroid gland	Assocaited abnormalities
**N70-1**	***TSHR***	c.141delC	p.I47fs	**P**	Hom	NA	NA	NA	aplasia	None
**N70-2**	***TSHR***	c.141delC	p.I47fs	**P**	Hom	NA	NA	NA	aplasia	None
**N71**	***TSHR***	c.C484G	p.P162A	**LP**	Het	0.00017	0.0001371	Reported [53]	hypoplasia	None
**N72-1**	***TSHR***	c.G902A	p.C301Y	US	Het	NA	0.000032	NA	Normal	None
**N72-2**	***TSHR***	c.G902A	p.C301Y	US	Het	NA	0.000032	NA	Normal	None
**N73**	***TSHR***	c.C1532T	p.T511M	US	ComHet	0.000033	0.000033	NA	Normal	None
	***TSHR***	c.T1697G	p.V566G	US		NA	NA	NA		
**N74**	***NKX2-1***	c.628_772del		**P**	Het	NA	NA	NA	hypoplasia	chorea
**N75**	***NKX2-1***	c.A1180G	p.T394A	US	Het	NA	NA	NA	aplasia	None
**N76**	***NKX2-5***	c.G676A	p.D226N	US	Het	NA	NA	NA	hypoplasia	None
**N77**	***PAX8***	c.A701G	p.E234G	US	Het	NA	0.000037	NA	hypoplasia	None
**N78**	***PAX8***	c.G440A	p.C147Y	US	Het	NA	NA	NA	hypoplasia	None
**N79**	***PAX8***	chr2:113973574_ 114036498del		**P**	Het	NA	NA	NA	hypoplasia	None

^**#**^ The Human Gene Mutation Database (HGMD® (http://www.hgmd.cf.ac.uk) [52]

*ExAC database (http://exac.broadinstitute.org) [36]

**^**gnomAD database (http://gnomad.broadinstitute.org/)

Pathogenicity: US, Uncertain significance; P, Pathogenic; LP, Likely pathogenic (pathogenicity rated according to ACMG guidelines [29], sequence variants rated as ‘benign’ or ‘likely benign’ were excluded from the analysis); NT, nucleotide; AA, amino acid; NA, not available; Het, heterozygous; ComHet, compound heterozygous; Hom, homozygous.

NCBI Reference Sequences (www.ncbi.nlm.nih.gov/nuccore): *TSHR*, NM_000369; *NKX2-1*, NM_001079668; *NKX2-5*, NM_004387; *PAX8*, NM_003466.

Frequency distribution of variants in genes associated with TD was as follows: *TSHR* 6.5% (6/92), *NKX2-1* 2.2% (2/92), *NKX2-5* 1.1% (1/92), *PAX8* 3.3% (3/92). In our study, a deletion with frameshift and 4 missense variants were detected in *TSHR* (Table 2). MLPA was conducted in the patients with a single heterozygous variant (N71, N72-1, N72-2) and showed no extended deletions or insertions. We found one deletion with frameshift and one missense variant in *NKX2-1* gene and one heterozygous missense variant in *NKX2-5* gene (Table 2). In three cases mutations in *PAX8* were detected (Table 2), 2 of which were missense variants, and in one patient (N79) an extended deletion in the gene was suspected by NGS and subsequently confirmed by MLPA. There were no mutations in *DOUXA2* or *FOXE1* genes.

Mutations in two genes were revealed in 8 patients (8.7%) (Table 3). The most frequent combinations of variants in DH genes were *TG* and *TPO* (3 cases). 2 cases showed a combination of variants in DH and TD genes: *TG* and *PAX8* (1 case), and *TSHR* and *DUOX2* in 1 case.

**Table 3:** Digenic mutations, characteristics and clinical manifestations.

Subjects	Gene	NT alteration	AA alteration	Pathogenicity	Zygosity	ExAC*	gnomAD^	HGMD^#^	Thyroid gland	Assocaited abnormalities
**N80**	***PAX8***	c.C74T	p.P25L	US	Het	NA	NA	NA	hypoplasia	None
**N80**	***TG***	c.C961T	p.R321X	**P**	Het	NA	NA	NA		
**N81**	***TG***	c.C6553T	p.R2185W	US	Het	NA	0.000048	NA	hypoplasia	None
**N81**	***TPO***	c.C208G	p.P70A	US	Het	0.00072	0.00086	NA		
**N82**	***IYD***	c.C818T	p.T273M	US	Het	0.00013	0.00011	NA	hypoplasia	None
**N82**	***TG***	c.G2977A	p.A993T	US	Het	0.00033	0.00039	NA		
**N83**	***DUOX2***	c.2895_2898del	p.S965fsX30	**P**	Het	0.0029	NA	Reported [46]	hypoplasia	None
**N83**	***TSHR***	c.G733A	p.G245S	US	Het	0.00014	0.00009	Reported [54]		
**N84**	***TG***	c.G455A	p.R152H	US	Het	0.00068	0.00073	NA	goiter	None
**N84**	***TPO***	c.C290G	p.S97X	**P**	Het	NA	NA	NA		
**N85**	***DUOX2***	c.A4603G	p.R1535G	US	Het	0.00027	0.00029	NA	goiter	None
**N85**	***TPO***	c.C962T	p.T321I	US	Het	NA	NA	NA		
**N86**	***DUOX2***	c.2895_2898del	p.S965fsX30	**P**	Hom	0.0029	NA	Reported [46]	goiter	None
**N86**	***SLC26A4***	c.G441A	p.M147I	US	Het	0.00051	0.0006	Reported [50]		
**N87**	***TG***	c.C4481T	p.P1494L	US	Het	0.00054	0.00047	NA	goiter	None
**N87**	***TPO***	c.G1450A	p.V484M	US	Het	NA	NA	NA		

^**#**^ The Human Gene Mutation Database (HGMD® (http://www.hgmd.cf.ac.uk) [52]

*ExAC database (http://exac.broadinstitute.org) [36]

^gnomAD database (http://gnomad.broadinstitute.org/)

Pathogenicity: US, Uncertain significance; P, Pathogenic; LP, Likely pathogenic (pathogenicity rated according to ACMG guidelines [29], sequence variants rated as ‘benign’ or ‘likely benign’ were excluded from the analysis); NT, nucleotide; AA, amino acid; NA, not available; Het, heterozygous; ComHet, compound heterozygous; Hom, homozygous.

NCBI Reference Sequences (www.ncbi.nlm.nih.gov/nuccore): *TPO*, NM_000547; *DUOX2*, NM_014080; *TG*, NM_003235; *SLC26A4*, NM_000441; *IYD*, NM_203395; *TSHR*, NM_000369; *PAX8*, NM_003466.

In the control group, 4 heterozygous missense variants (7.1%) with uncertain significance were identified (Table 4). In comparison with the control group, the mutation rate in patients with CH was significantly higher (Pearson's χ2 (p<0.01), odds ratio = 7.9, confidence interval 2.7–22.6.

**Table 4:** Control group.

Subjects	Gene	NT alteration	AA alteration	Pathogenicity	Zygosity	ExAC*	gnomAD^	HGMD^#^
**C1**	*DUOX2*	c.C4632G	p.H1544Q	US	Het	NA	NA	NA
**C2**	*IYD*	c.A281G	p.Y94C	US	Het	0.000025	0.00004	NA
**C3**	*SLC26A4*	c.C1232G	p.A411G	US	Het	NA	NA	NA
**C4**	*TG*	c.A6853G	p.N2285D	US	Het	NA	NA	NA

^**#**^ The Human Gene Mutation Database (HGMD® (http://www.hgmd.cf.ac.uk) [52]

*ExAC database (http://exac.broadinstitute.org) [36]

**^**gnomAD database (http://gnomad.broadinstitute.org/)

Pathogenicity: US, Uncertain significance; P, Pathogenic; LP, Likely pathogenic (pathogenicity rated according to ACMG guidelines [29], sequence variants rated as ‘benign’ or ‘likely benign’ were excluded from the analysis); NT, nucleotide; AA, amino acid; NA, not available; Het, heterozygous; ComHet, compound heterozygous; Hom, homozygous.

NCBI Reference Sequences (www.ncbi.nlm.nih.gov/nuccore): *DUOX2*, NM_014080; *TG*, NM_003235; *SLC26A4*, NM_000441; *IYD*, NM_203395.

In the group of patients with variants in one of DH genes, the most frequent pattern according to the ultrasound was hypoplasia of the thyroid gland, 32.0% (23/72), different forms of goiter, including multinodular, were identified in 26.4% (19/72), 11.1% of cases (8/72) had normal volume of the thyroid according to WHO criteria [32,33]. Thyroid aplasia was revealed in 6.9% (5/72) and ectopia in 1.4% (1/72). We were unable to obtain data of the thyroid size in 22.2% (16/72) of cases. The majority of cases with variants in TD genes showed hypoplasia or aplasia of the thyroid gland (75.0%, 9/12), three patients with variants in *TSHR* gene had normal thyroid volume.

## Discussion

Recent studies have shown that the frequency of gene defects associated with CH is substantially higher than previously estimated, and ranges from 33,0% to 61,5% [24–28]. However, these studies were limited by either the number of genes selected for analysis [27,28] or the number of the patients included in the study [24,25,27]. In addition, relatively soft filtering criteria for selection of pathogenic variants have been reported, allowing for MAF as high as 0.01 [24,26–28]. In the current study, using an NGS panel for 12 CH genes associated both with thyroid dysgenesis and dyshormonogenesis disorders, we have assessed the spectrum of gene defects in Russian subjects with severe CH, regardless of the thyroid anatomy findings. We have used more stringent criteria for selection of potentially pathogenic sequence variants, which were based on the recent ACMG guidelines [29]. As the result, from the analysis were excluded all single nucleotide variants with MAF greater than 0.001. For instance, P303R variant in *DUOX2* gene (rs151261408, MAF = 0.01), rated as likely pathogenic by Lof et al [24], was found in our cohort in 24 of 243 subjects (not shown). This variant previously shown to have no effect on DUOX2 function by *in vitro* experiments [34] is classified as BS1, BS3 (benign) by ACMG rating [29] and excluded from analysis.

The results of the study demonstrate the genetic heterogeneity of CH and a high incidence of cases with pathogenic or potentially pathogenic variants in one of the CH candidate genes (37.9%), both in patients with thyroid dysgenesis and goiter and normal size of the gland. In general, according to Exome Aggregation Consortium (ExAC) data (http://exac.broadinstitute.org/), the majority of CH genes (DH genes, in particular) show higher than expected variant counts (low intolerance to variation) [35]. To evaluate the chances of having a variant in one of CH genes in subjects without CH we have sequenced the candidate genes in 56 subjects with normal thyroid function and demonstrated a significantly lower rate of variants compared to the CH group (OR = 7.9, p<0.01).

Moreover, according to the results of our study, the most frequent findings in severe CH were variants in DH genes 84.8% (78/92), while only 13.1% (12/92) of cases were associated with variants in TD genes, which contradicts to the expected distribution of etiological forms based on the results of ultrasound and scintigraphy [2,3]. The more prevalence of mutations in DH genes compared to TD genes have been also reported in other NGS-based studies [24,25,27,28].

The majority of TD disorders were originally described as autosomal recessive, however, a large proportion of variants identified in our study, both using targeted NGS and additional screening of extended deletions by MLPA, were heterozygous. Existence of additional mutation in non-coding regions of the studied genes can not be completely ruled out. Another possible explanation could be non-Mendelian mechanisms of inheritance, such as autosomal monoallelic expression (AME) [36–38]. Initially, autosomal monoallelic expression of the mutant allele was described for *TPO* gene [36]. The subsequent study by Magne et al. demonstrated AME on average for 22 genes [16–32] expresses in the thyroid [39]. Monoallelic mutations in TD genes in subjects with CH have been reported by others [25,26]. Fan et al. identified 9 cases with mutations in *TG* gene, all of which were heterozygous [25].

Another unexpected finding was the absence of goiter in some patients with defects in DH genes. A similar observation has been made by Kühnen et al. who detected a homozygous missense mutation in *SLC26A4* gene in patients with thyroid hypoplasia [40]. The authors suggested a role of severe postnatal iodine deficiency as a possible explanation of this phenomenon [41]. Another reason for the absence of enlargement of the thyroid can be anti-goitrogenic effect of levothyroxine.

Similar to some previous reports [24–28,41], we identified patients with digenic mutations. The development of hypothyroidism in such cases is explained by synergistic heterozygosity, so the presence of heterozygous mutations in several genes can lead to cross-loss of enzyme activity [42]. In our study digenic mutations were found in 8 patients. Interestingly, goiter in this group was identified only in patients with 2 mutations of DH genes, while patients with mutations both in DH and TD genes showed a decrease in the volume of the gland.

In summary, a targeted next generation sequencing in patients with severe CH revealed potentially pathogenic sequence variants in more than a third of the cases, with a preponderance of those in genes associated with thyroid dyshormonogenesis.

## Supporting information

S1 Fig Sanger confirmation of sequence variants identified by NGS. A) *TPO* c.1181_1182insCGGC; B) *TPO* c.1851delC; C) *TPO* c.G1581T; D) *TPO* c.G1994A; E) *TPO* c.G2017A; F) *TPO* c.G1042A; G) *TPO* c.667_669delGAT; H) *TPO* c.2422delT; I) *TPO* c.A719T; J) *DUOX2* c.2895_2898del; K) *DUOX2* c.A4637G.
(TIF)

S2 Fig Sanger confirmation of sequence variants identified by NGS. A) *DUOX2* c.C1126T; B) *DUOX2* c.C1294T; C) *DUOX2* c.C3250T; D) *DUOX2* c.C3970T; E) *DUOX2* c.G1040A; F) *DUOX2* c.T1366C; G) *TG* c.C2338A; H) *TG* c.G2977A; I) *SLC5A5* c.C1906T; J) *SLC5A5* c.469delA; K) *SLC26A4* c.A736C; L) *SLC26A4* c.G441A.
(TIF)

S3 Fig Sanger confirmation of sequence variants identified by NGS. A) *SLC26A4* c.G2219T; B) *IYD* c.C448T; C) *TSHR* c.141delC; D) *TSHR* c.C484G; E) *TSHR* c.G902A; F) *TSHR* c.C1532T; G) *NKX2-1* c.628_772del; H) *NKX2-1* c.A1180G; I) *NKX2-5* c.G676A; J) *PAX8* c.A701G; K) *PAX8* c.G440A; L) *PAX8* c.C74T.
(TIF)

S4 Fig Sanger confirmation of sequence variants identified by NGS. A) *TG* c.C961T; B) *TG* c.C6553T; C) *TSHR* c.G733A; D) *TG* c.G455A; E) *DUOX2* c.A4603G; F) *TG* c.C4481T; G) *TPO* c.G1450A; H) *TPO* c.C443T; I) *TPO* c.T391C.
(TIF)

S5 Fig MLPA result. *PAX8* chr2:113973574_114036498del.
(TIFF)
