# Supplementary figures and images for: High frequency of mutations in 'dyshormonogenesis genes' in severe congenital hypothyroidism
Source: PLoS One. 2018 Sep 21;13(9):e0204323. doi: 10.1371/journal.pone.0204323 (PMC6150524; doi:10.1371/journal.pone.0204323)

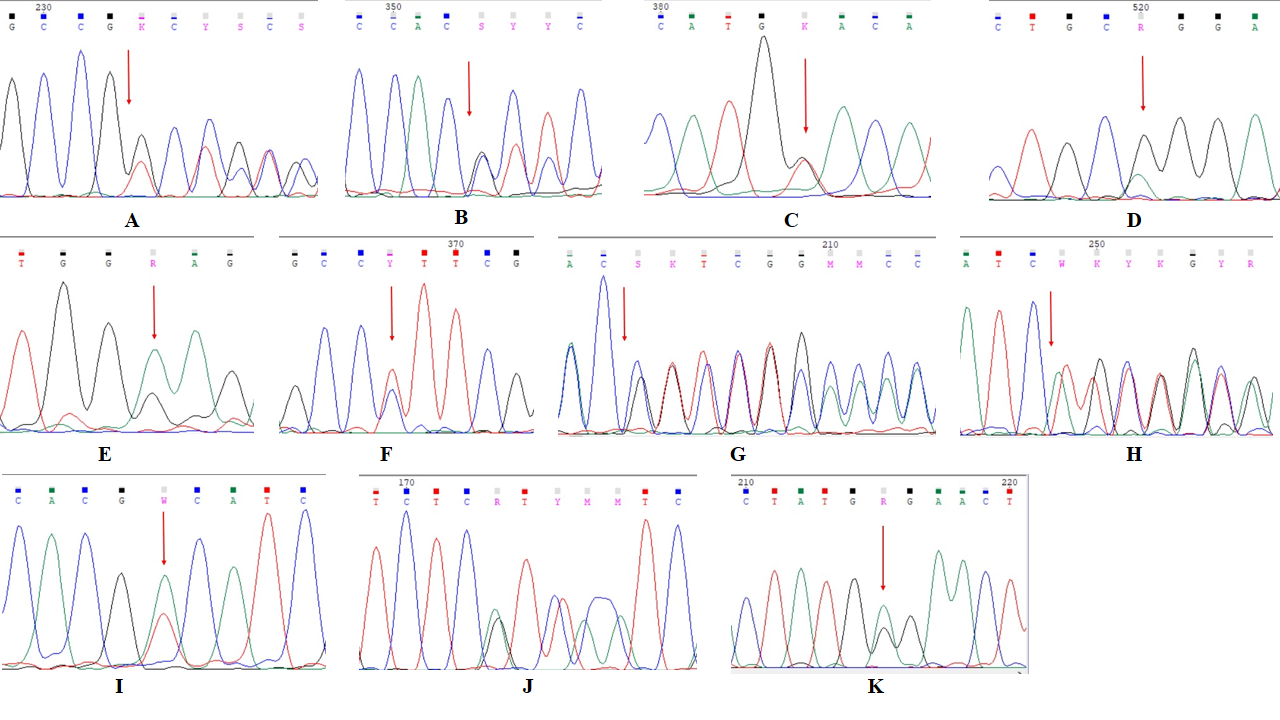

Supplement: S1 Fig — A) TPO c.1181_1182insCGGC; B) TPO c.1851delC; C) TPO c.G1581T; D) TPO c.G1994A; E) TPO c.G2017A; F) TPO c.G1042A; G) TPO c.667_669delGAT; H) TPO c.2422delT; I) TPO c.A719T; J) DUOX2 c.2895_2898del; K) DUOX2 c.A4637G. (TIF) [file pone.0204323.s001.tif]

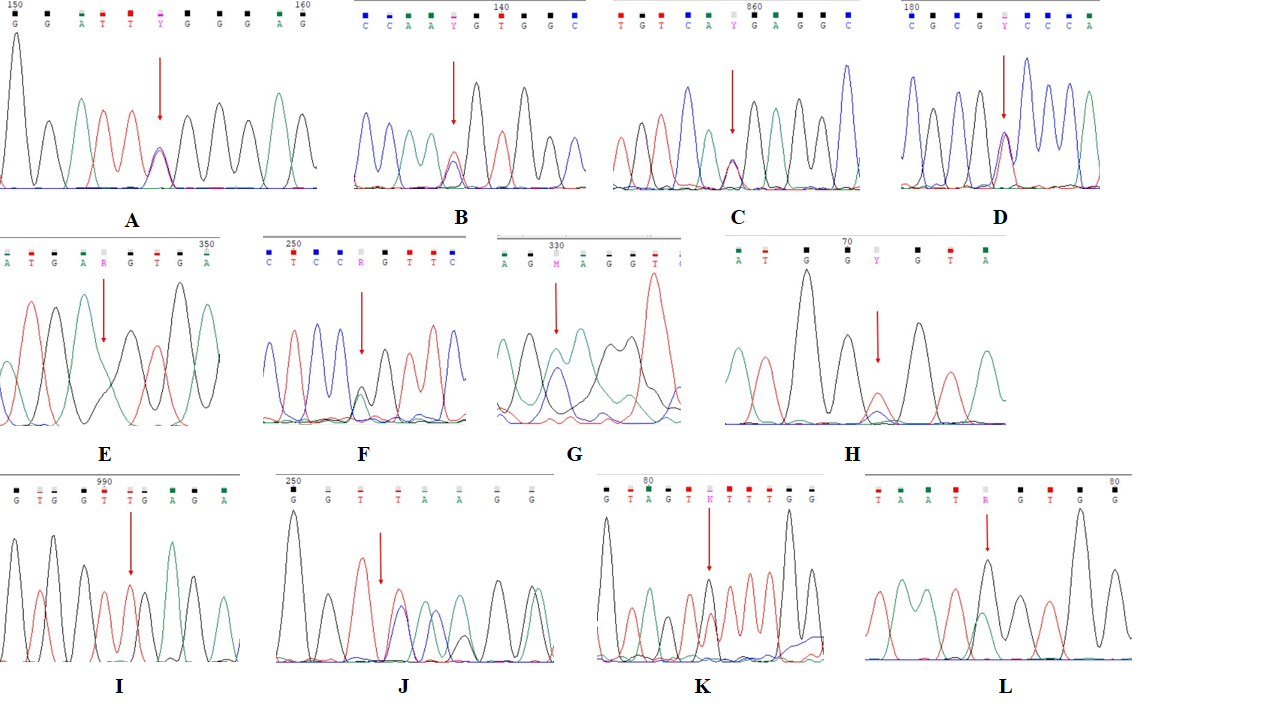

Supplement: S2 Fig — A) DUOX2 c.C1126T; B) DUOX2 c.C1294T; C) DUOX2 c.C3250T; D) DUOX2 c.C3970T; E) DUOX2 c.G1040A; F) DUOX2 c.T1366C; G) TG c.C2338A; H) TG c.G2977A; I) SLC5A5 c.C1906T; J) SLC5A5 c.469delA; K) SLC26A4 c.A736C; L) SLC26A4 c.G441A. (TIF) [file pone.0204323.s002.tif]

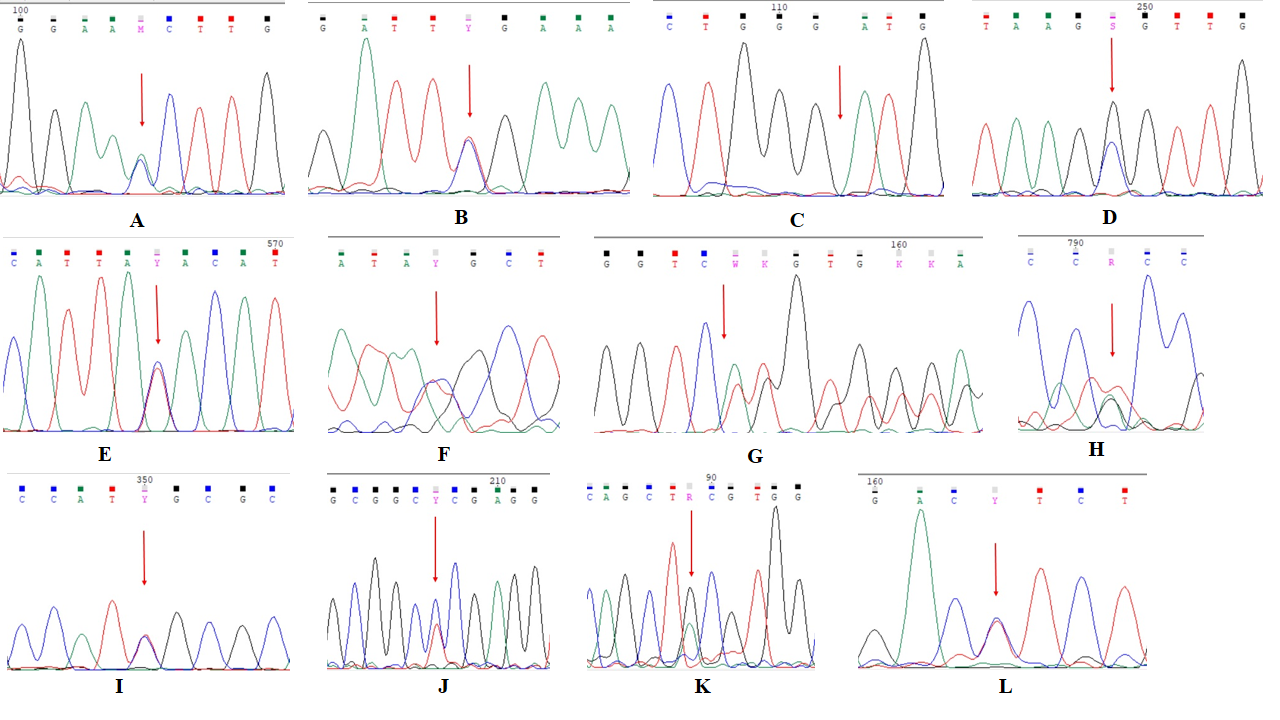

Supplement: S3 Fig — A) SLC26A4 c.G2219T; B) IYD c.C448T; C) TSHR c.141delC; D) TSHR c.C484G; E) TSHR c.G902A; F) TSHR c.C1532T; G) NKX2-1 c.628_772del; H) NKX2-1 c.A1180G; I) NKX2-5 c.G676A; J) PAX8 c.A701G; K) PAX8 c.G440A; L) PAX8 c.C74T. (TIF) [file pone.0204323.s003.tif]

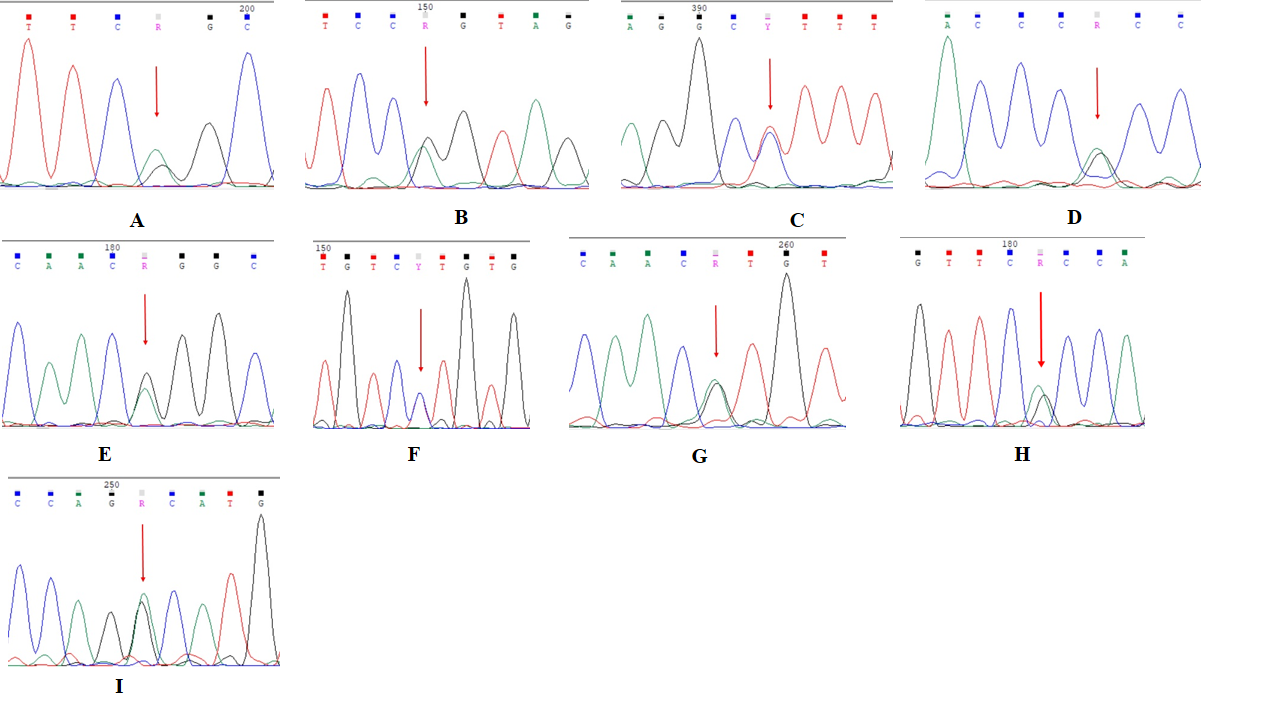

Supplement: S4 Fig — A) TG c.C961T; B) TG c.C6553T; C) TSHR c.G733A; D) TG c.G455A; E) DUOX2 c.A4603G; F) TG c.C4481T; G) TPO c.G1450A; H) TPO c.C443T; I) TPO c.T391C. (TIF) [file pone.0204323.s004.tif]

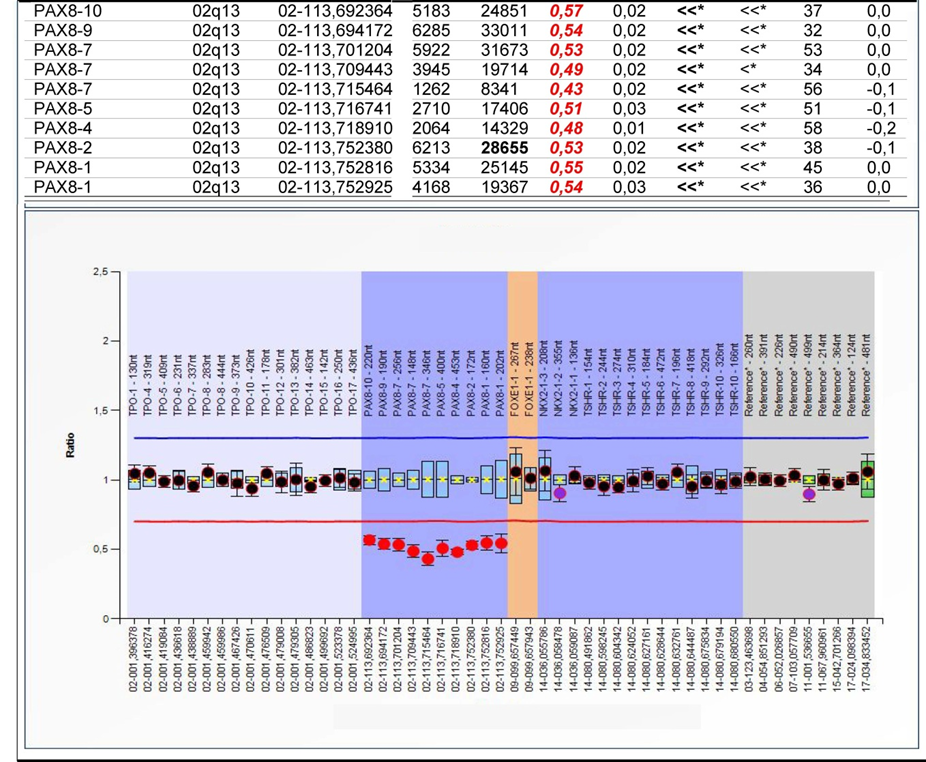

Supplement: S5 Fig — PAX8 chr2:113973574_114036498del. (TIFF) [file pone.0204323.s005.tiff]
